# SARM1 Suppresses Axon Branching Through Attenuation of Axonal Cytoskeletal Dynamics

**DOI:** 10.3389/fnmol.2022.726962

**Published:** 2022-02-21

**Authors:** Andrea Ketschek, Sabrina M. Holland, Gianluca Gallo

**Affiliations:** ^1^Shriners Hospitals Pediatric Research Center, Lewis Katz School of Medicine, Temple University, Philadelphia, PA, United States; ^2^Department of Neural Sciences, Lewis Katz School of Medicine, Temple University, Philadelphia, PA, United States

**Keywords:** axon, SARM, actin, microtubule, cortactin, drebrin

## Abstract

Axon branching is a fundamental aspect of neuronal morphogenesis, neuronal circuit formation, and response of the nervous system to injury. Sterile alpha and TIR motif containing 1 (SARM1) was initially identified as promoting Wallerian degeneration of axons. We now report a novel function of SARM1 in postnatal sensory neurons; the suppression of axon branching. Axon collateral branches develop from axonal filopodia precursors through the coordination of the actin and microtubule cytoskeleton. *In vitro* analysis revealed that cultured P0-2 dorsal root ganglion sensory neurons from a SARM1 knockout (KO) mouse exhibit increased numbers of collateral branches and axonal filopodia relative to wild-type neurons. In SARM1 KO mice, cutaneous sensory endings exhibit increased branching in the skin *in vivo* with normal density of innervation. Transient axonal actin patches serve as cytoskeletal platforms from which axonal filopodia emerge. Live imaging analysis of axonal actin dynamics showed that SARM1 KO neurons exhibit increased rates of axonal actin patch formation and increased probability that individual patches will give rise to a filopodium before dissipating. SARM1 KO axons contain elevated levels of drebrin and cortactin, two actin regulatory proteins that are positive regulators of actin patches, filopodia formation, and branching. Live imaging of microtubule plus tip dynamics revealed an increase in the rate of formation and velocity of polymerizing tips along the axons of SARM1 KO neurons. Stationary mitochondria define sites along the axon where branches may arise, and the axons of SARM1 KO sensory neurons exhibit an increase in stationary mitochondria. These data reveal SARM1 to be a negative regulator of axonal cytoskeletal dynamics and collateral branching.

## Introduction

Morphogenesis of axons is a fundamental aspect of neurodevelopment. Although each neuron forms a single axon, the process of axon branching allows a single axon to form numerous connections with target neurons often in disparate regions of the nervous system (Lewis et al., 2013; Kalil and Dent, 2014; Menon and Gupton, 2018). A major form of axon branching is collateral axon branching wherein an axon gives rise to a new branch from along its shaft independent of the growth cone. Axon collateral branching is also of importance in the context of the response of the nervous system to injury, wherein branching induced in the injured scenario can be either adaptive or maladaptive (Onifer et al., 2011). Thus, understanding the cellular mechanisms of axon branching is of broad significance.

Collateral axon branching involves coordination of the axonal cytoskeleton (reviewed in Gallo, 2011; Kalil and Dent, 2014; Pacheco and Gallo, 2016; Armijo-Weingart and Gallo, 2017). The first step in collateral axon branching is formation of actin filament (F-actin)-dependent filopodia. Transient meshworks of dynamic F-actin along an axon, termed actin patches, serve as precursors to filopodia formation (reviewed in Gallo, 2011; Kalil and Dent, 2014). Promotion of axon branching by NGF involves upregulation of the rate of actin patch formation and, subsequently, formation of filopodia along axons (Ketschek and Gallo, 2010; Spillane et al., 2012). For a filopodium to mature into a branch, it must be invaded by an axonal microtubule. Stabilization of a microtubule within a filopodium allows the filopodium to mature into a branch, as it reorganizes its actin filament cytoskeleton, developing a small growth cone at its tip and increasing levels of microtubules. The cytoskeletal mechanism of axon branching has been shown to involve multiple actin and microtubule regulatory proteins, and it is strictly dependent on the coordination of actin and the cytoskeleton of microtubules (Pacheco and Gallo, 2016; Armijo-Weingart and Gallo, 2017). In the context of branching induced or suppressed by extracellular signals, the axis of PI3K signaling has been shown to have important roles in promoting the actin filament component of branching, including axonal translation of actin regulatory proteins important for branching, and PI3K signaling is impaired by branching inhibitory signals (reviewed in Gallo, 2011). Axonal organelles also have important roles in axon branching (reviewed in Winkle et al., 2016). Stationary mitochondria denote sites along an axon where axonal filopodia and, subsequently, branches develop (Courchet et al., 2013; Spillane et al., 2013; Tao et al., 2014; Sainath et al., 2017a). Increasing the motility of mitochondria and, thus, decreasing stationary mitochondria suppresses branching (Courchet et al., 2013); similarly, inhibiting the respiration of mitochondria suppresses branching (Spillane et al., 2013). Axonal mitochondria undergo transport into branches after their initial development and presumably power their continued extension (Spillane et al., 2013; Armijo-Weingart et al., 2019).

The TIR domain adaptor protein sterile alpha and TIR motif containing 1 (SARM1)/Myd-885 was initially considered to mediate aspect of immune function (Panneerselvam and Ding, 2015) and subsequently revealed to promote cell intrinsic axon Wallerian degeneration (Osterloh et al., 2012). Roles of SARM1 have also been described in neuronal cell death in toxic scenarios (Kim et al., 2007; Hou et al., 2013; Mukherjee et al., 2013; Summers et al., 2014). Knock down of SARM1 in hippocampal neurons results in decreased dendrite development and shorter axons (Chen et al., 2011). In neurons, SARM1 is mitochondrially targeted through an N-terminus-targeting sequence, although mitochondrial targeting is not required for its promotion of axon degeneration (Gerdts et al., 2013). During the course of preliminary studies investigating the role of SARM1 in the regulation of microtubule loss during Wallerian degeneration, we noticed that cultured sensory neurons from SARM1 knockout (KO) mice exhibited more complex axonal morphologies than wild-type counterparts. Here, we report that in sensory neurons, SARM1 KO results in increased axon branching due to the promotion of cytoskeletal dynamics underlying the formation of axonal filopodia and axonal microtubules plus tip dynamics, unveiling a negative regulatory role of SARM1 in the regulation of the axonal F-actin cytoskeleton.

## Materials and Methods

### Culturing and Animals

The animal study was reviewed and approved by Temple University School of Medicine Institutional Animal Care and Use Committee. For culturing, pups were obtained from mating pairs of the same genotype: SARM1 KO (−/−) mice (Kim et al., 2007) and wild-type background C57BL/6. Mating pairs of SARM1 KO (−/−) and SARM1 wild type (WT, + / +) were initially derived from mating heterozygous (±) SARM1 KO pairs and genotyping the progeny, and then mating within genotype thereafter for subsequent mating.

Postnatal day (P) P0-2 mice were placed on ice for 10 min, and 1.5 DRGs were dissected per coverslip and placed in CMF-PBS + 5 mg/ml dispase (#11534200; Roche, Branchburg, NJ, United State) in 37° water bath for 15 min. A pellet was spun down for 30 s, and the supernatant was removed and replaced with trypsin (#MT25005CI; Thermo Fisher Scientific, Hampton, NH, United States) and placed back in the water bath. Following 10 min in trypsin, the pellet was spun down for 1 min, the supernatant was removed, and cells were re-suspended and triturated in 5 ml F12HS10. The cells were centrifuged for 5 min, the supernatant was removed, and the cells were re-suspended in a culturing medium containing F12H + 50 ng/ml NGF (#256-GF; R&D, Minneapolis, MN, United States). A 500-ml culturing medium containing dissociated cells was added to each coverslip or 300 μl for each “videodish” (coverslip affixed to the bottom of a plastic Petri dish with a hole drilled in the center). The coverslips were coated using polylysine (100 μg/ml (Cat # P9011; Sigma, St. Louis, MO, United States) and laminin (25 μg/ml, Cat # 23017-015; Life Technologies, Carlsbad, CA, United States). Chondroitin sulfate proteoglycans (CSPG) substrata were prepared as previously described using embryonic day 14 chicken embryo CSPG mixtures (as in Silver et al., 2014; Sainath et al., 2017a,b).

The media used were as previously detailed (Lelkes et al., 2005). Briefly, the F12HS10 dissection medium consisted of Hams F12 1X with L-glutamine (#MT10080CV; Thermo Fisher Scientific, Hampton, NH, United States) containing 10% fetal bovine serum (#MT35011CV; Thermo Fisher Scientific, Hampton, NH, United States) and 1% HEPES 1M (#BP299-100; Thermo Fisher Scientific, Hampton, NH, United States). The F12H culturing medium consisted of F12 nutrient mix (#21700075; Thermo Fisher Scientific, Hampton, NH, United States) dissolved in 1 L of distilled water. The following ingredients were supplemented into the F12H culturing medium and were listed as final concentrations, 1% Hepes 1M (#BP299-100; Thermo Fisher Scientific, Hampton, NH, United States), 1% PSF (#BW17745E; Thermo Fisher Scientific, Hampton, NH, United States), 1% L-glutamine 200 mM (#MT25005CI; Thermo Fisher Scientific, Hampton, NH, United States), 4% sodium pyruvate 100 mM (#11360070; Thermo Fisher Scientific, Hampton, NH, United States). The F12H culturing medium was also supplemented with an additive cocktail containing the following ingredients: phosphocreatine (#P-7936; Sigma, St. Louis, MO, United States), apo-transferrin (#T-2252; Sigma, St. Louis, MO, United States), sodium selenate (#S-8295; Sigma, St. Louis, MO, United States), progesterone (#P-8783; Sigma, St. Louis, MO, United States), and insulin (#I-5500; Sigma, St. Louis, MO, United States).

### Transfection and Plasmids

For transfection of plasmids into neurons, 30 mouse DRGs were dissociated as described above, and after F12HS10 was removed, neurons were suspended in a 100-μl nucleofector solution (Cat# VPG-1002; Lonza) and gently resuspended through trituration. The DRG cell suspension was transferred to a nucleofector cuvette containing 15 μg of plasmid DNA, and electroporated using Amaxa Nucleofector (program G-13). The electroporated solution was then immediately transferred to a tube containing the F12H medium as described above prior to plating. The mitochondrially targeted GFP expression plasmid used was previously described in Spillane et al. (2013), mCherry-β-actin as in Spillane et al. (2013), SARM1 (Chen et al., 2011; Addgene, #50707) was subcloned into pEGFP-N1 (Clontech), GFP-EB3 was as in Ketschek et al. (2007).

### Immunocytochemistry

The following protocol was used for all staining purposes. Fixed cultures were blocked prior to each primary antibody application for 30 min using PBS supplemented with 10% goat serum (#G9023; Sigma, St. Louis, MO, United States) and 0.1% triton X-100 (#9002-93-1; Sigma, St. Louis, MO, United States) (GST). All antibodies were applied using GST. Primary and secondary antibody staining was performed at room temperature for 45 min. Washing in PBS was performed after primary and secondary staining. Prior to the coverslips being mounted with Vectashield (#H-1000; Vector), they were washed 3 × with distilled water. Within a given experiment, replicates were initially fixed and stored in a blocking solution at 4^°^C until control and experimental samples could be stained in parallel on the same day prior to subsequent imaging.

To visualize cortactin (1:250, # AB11065, RRID:AB_297716; Abcam, Cambridge, United Kingdom) and Akt phosphorylated at T308 (pAkt; 1:100, #9275, RRID:AB329828; Cell Signaling Technology, Danvers, MA, United States), cultures were fixed with a final concentration of 4% PFA (#15710; EMS) and 5% sucrose (#S5-500; Thermo Fisher Scientific, Hampton, NH, United States). Secondary staining was performed using goat-anti-rabbit FITC (1:200, #F9887, RRID:AB_259816; Sigma, St. Louis, MO, United States). Actin filaments were stained using rhodamine phalloidin (1:20, #R415, RRID:AB_2572408; Invitrogen, Waltham, MA, United States). To visualize drebrin (1:100, M2F6, RRID:AB_299034; Abcam, Cambridge, United Kingdom), cultures were fixed with a final concentration of 0.25% glutaraldehyde (#16300; EMS). Secondary staining was performed using goat-anti-mouse TRITC (1:400, #T5393, RRID:AB_261699; Sigma, St. Louis, MO, United States). Actin filaments were stained using DyLight 488 Phalloidin (1:20, Cat# 21833, RRID:AB_2532155; Thermo Fisher Scientific).

To determine the ratios of acetylated and tyrosinated tubulin compared to total tubulin, cultures were simultaneously fixed and extracted using 0.2% gluteraldehyde and 0.1% triton X-100 in a PHEM buffer (10 mM MES, 138 mM KCl, 3 mM MgCl, and 2 mM EGTA), followed by 15-min incubation with 2 mg/ml sodium borohydride (#Ac189301000; Thermo Fisher Scientific, Hampton, NH, United States) in CMF-PBS. Samples were stained with anti-acetylated tubulin (clone 6-11B-1) (1:100, #T6793, RRID:AB_477585; Sigma, St. Louis, MO, United States) or anti-tyrosinated tubulin (Tub1A2) (1:400, #T9028, RRID:AB_261811; Sigma, St. Louis, MO, United States). Secondary staining was performed with goat-anti-mouse TRITC (1:400, #T5393, RRID:AB_261699; Sigma, St. Louis, MO, United States). The samples were then blocked with 10% mouse serum for 20 min and counterstained with DM1A-FITC anti-α tubulin (1:100, #F2168, RRID:AB_476967; Sigma, St. Louis, MO, United States) to visualize total microtubule intensity.

### Imaging

All imaging was performed using a Carl Zeiss 200M microscope equipped with an Orca ER camera (Hamamatsu, Shizuoka, Japan). Live cultures were placed in a heated microscope stage (Zeiss temperable insert P with objective heater) and kept at constant 39°C. Zeiss Live and fixed cultures were observed using a 100 ×, 1.3 numerical aperture objective for both phase and fluorescence microscopy. A time-lapse and quantitative analysis was performed using the interactive measurement module of the AxioVision Software. To visualize mitochondria, Mitotracker Red (0.05 nM, # M22425; Invitrogen, Waltham, MA, United States) was used according to the directions of the manufacturer. Cultures were incubated with a dye for 30 min, washed with a medium, and imaged 20 min following wash cycles. Acquisition rate was every 6 s for 10 min. For actin patch formation observation, cells were transfected with mCherry-β-actin. Images were taken every 6 s for 6 min. To visualize the tips of polymerizing microtubules, cells were transfected with GFP-EB3, and images were taken every 6 s for 5 min. Minimal light from a 100-W bulb was used, and 2 × 2 camera binning was performed to increase sensitivity under low light exposure conditions.

### Morphometrics

For analysis of the number of branches per unit length of axon in the stained samples to reveal microtubules and actin filaments, we followed the criteria previously defined in Spillane et al. (2013). Briefly, a collateral branch was defined as containing detectable stained microtubules and distally polarized actin filament cytoskeleton to differentiate from axonal filopodia that contained microtubules but retained the characteristic uniform distribution of actin filaments along their shaft. For analysis of the number of branches along the distal 50-micron termini of PGP9.5 labeled nerve fibers located in the stratum spinosum, we counted the number of tips visualized in maximal projections of one-micron Z-stacks associated with individual fibers and subtracted one, as one would reflect the terminus of the fiber and not an additional branch. Growth cone area and the number of filopodia were measured and previously described (Jones et al., 2006) from images of the actin filament cytoskeleton of growth cones. Briefly, the area of the growth cones is measured from the “neck” of the growth cones to the distal most lamellipodial protrusion excluding filopodia. If a growth cone does not exhibit lamellipodia but only filopodia, or is collapsed, then area measurement is performed from the distal 5 microns of the axon shaft, representative of the mean length of growth cones that have lamellipodia. All length and area measurements were performed using the AxioVision software (Zeiss, Oberkochen, Germany).

### Analysis of Length and Density of Mitochondria

Mitochondrial length and density were measured from mitochondria labeled with MitoTracker Red as described above. For detailed consideration of methodological issues related to these metrics along sensory axons (see Armijo-Weingart et al., 2019). Mitochondrial length was determined by line measurements spanning the two ends of the mitochondrion. Although most axonal mitochondria are linear, if bent, then segmented line analysis was performed to track the curvature of the mitochondria to obtain a full-length measurement. Mitochondria in clusters consisting of two or more are not amenable to the analysis described above and were excluded from measurements of length and density in all imaging sets. The lengths of the axon segments containing clusters of mitochondria were thus also excluded from the final determination of mitochondria density (mitochondria/unit length of axon).

### Analysis of Actin Patches and EB3 Comets

Actin patches were analyzed following criteria and methods previously detailed (Loudon et al., 2006; Ketschek and Gallo, 2010; Spillane et al., 2012; Sainath et al., 2017a; Armijo-Weingart et al., 2019). Briefly, patch formation rate per unit length and time were determined by counting instances of patch formation as determined by the local intensity of mCherry-actin increasing above the cytoplasmic baseline, intensity, and size until dissipating back to baseline cytoplasmic intensity level. Duration of patch was the number of seconds from when a patch became detectable to when it dissipated. The proportion of patches giving rise to filopodia was determined by considering all patches that formed and whether they gave rise to a filopodial tip.

EB3 comet dynamics were imaged and analyzed as previously described (Ketschek et al., 2007; Spillane et al., 2012). Briefly, as with actin patches, the formation of a comet was defined as a localized increase in GFP-EB3 intensity increase above the cytoplasmic baseline and the comets formed be unit time and length of axon thus determined. Duration of a comet was defined as the time from when a comet became visible above the baseline level to when it dissipated and no longer visible above the baseline. The average velocity of comet advance during the lifespan of a comet was determined by dividing the total distance advanced by the duration. For the measurements of comets, only comets that advanced anterogradely in axons were scored and those that entered growth cones were not counted, as for these parameters the analysis focused solely on axons. For determination of velocity and duration, the first 10 comets formed per axon were scored. The mean number of comets present per unit length of axon or within the growth cone was assessed by counting the number of visible comets at-30-s intervals within an axon or a growth cone. All distance measurements were performed using the AxioVision software (Zeiss, Oberkochen, Germany).

### Quantification of Staining Intensity

All immunocytochemical samples within an experiment were processed in parallel prior to imaging and imaged during the same imaging session using identical imaging parameters and the microscopic setup described in the imaging section. Quantification of total microtubule intensity, ratios of tyrosinated and acetylated tubulin, and levels of cortactin, drebrin, and pAkt in the axons was performed by background subtraction as previously described (Jones et al., 2006; Spillane et al., 2012; Ketschek et al., 2016). Briefly, regions of interest (ROIs) encompassing the distal 50 μm of axon shafts but excluding axonal protrusions and growth cones were established along the length ranges of the axons described in the results. The same ROI was then used to perform background measurements by moving it off the axon and onto the substratum not containing cells. The background measurement was subtracted from the axonal measurement.

### Immunohistochemistry and Intra-Epidermal Nerve Fiber Density and Branching Analysis

Skin biopsies were obtained from the footpad of age-matched adult mice. Biopsy samples were processed for immunohistochemistry, and Intra-Epidermal Nerve Fiber Density (IENFD) was determined by three independent observers as described in Himeno et al. (2011) from thirty-micrometer sections imaged using a 20 × objective. Sections were stained with an anti-PGP9.5 antibody (# PA5-29012, 1:250; RRID:AB_2546488; Thermo Fisher Scientific, Hampton, NH, United States) and the secondary used was goat anti-rabbit FITC (1:200, #F9887; Sigma, St. Louis, MO, United States). Skin was fixed in 2% paraformaldehyde (vol/vol) for 3 h at 4°C. Following fixation, the tissue was washed with 0.1 M PBS 3 times (5 min/wash). The tissue was cryoprotected in 20% sucrose (wt/vol; 24–36 h) and sectioned with a cryostat. For immunostaining, 30-μm sections were cut to and placed in 0.1 M phosphate buffer. Sections were incubated in blocking solution for 1 h at room temperature (10% FBS, 0.1% Triton X-100 in PBS) and then with primary antibody overnight at 4°C on a shaker for 24–36 h. The primary antibody was washed 3 times (5 min/wash), followed by incubation with a secondary antibody for 2 h. The secondary antibody was washed as with the primary, and following 2 washes with dH_2_O, sections were mounted onto coverslips. For analysis of branching along the termini of individual sensory fibers in the sections, the sections were imaged at 20x and Z-stacks were obtained (using 1 micron z-intervals) and then maximal projections were generated form the Z-stacks using Image J. Branch number analysis was performed by counting the number of tips along the distal 50 microns of each nerve fiber and subtracting one (as reflective of the terminus of the axon itself). All analyses of the sections were performed blind to the genotype.

### Statistical Analysis

All the statistical analyses were performed using Instat, and graphical data representation was performed using the Prism (both from GraphPad Software Inc., San Diego, CA, United States). The normality of data distributions was determined through Kolmogorov and Smirnov test. For presentation purposes, data sets wherein all the comparison groups were normally distributed are presented using mean and standard error of the mean (SEM). Data sets wherein one or more comparison groups exhibited a non-normal distribution are presented as individual data points and the median is presented. Comparisons of two groups were performed either by Welch *t*-test or Mann-Whitney test depending on the normalcy of the data sets. Multiple comparison *post hoc* tests were performed by Bonferroni and Dunn’s test for parametric and non-parametric analyses, respectively. Categorical data sets are presented as percentages within category, but statistical analysis was performed on raw categorical data by Fisher exact test to compare 2 × 2 contingency tables, and chi squared test for independence on larger tables. For presentation, data sets that were determined to be normally distributed are presented using the relevant descriptive statistics of mean and SEM. Non-normal data sets are presented using dot plots to allow for assessment of the shape of the distribution, or histograms for distributions of the number of branches along distal axons. Sample sizes (n) denote the total number of independent observations in the data sets obtained from replicates, as detailed below.

### Study Design

For *in vitro* immunostaining experiments, 3 mice/genotype were used to produce cultures on 3 separate days, with sampling distributed evenly across sessions. As noted above, for immunostaining and imaging, the cultures were processed in parallel prior to imaging and imaged during the same imaging session using identical imaging parameters and the microscopic setup described in the Imaging section. An ANOVA (parametric or non-parametric, determined by the assessment of distributions as described above) was performed for the relevant metric in the specific experiment within genotype across replicates to assess if differences could be discerned among replicates. In the absence of detected differences among the replicates, individual data points from the replicates were merged into a collective data set for each genotype within the experiment. The sizes of samples presented, thus, reflect the total number of data points obtained for a given metric from the data merged from the replicates. For live imaging, each axon was sampled from a separate culture spread across the replicates.

## Results

### Axons of SARM1 Knockout Sensory Neurons Exhibit Increase in Number of Axon Collateral Branches

**FIGURE 1:**
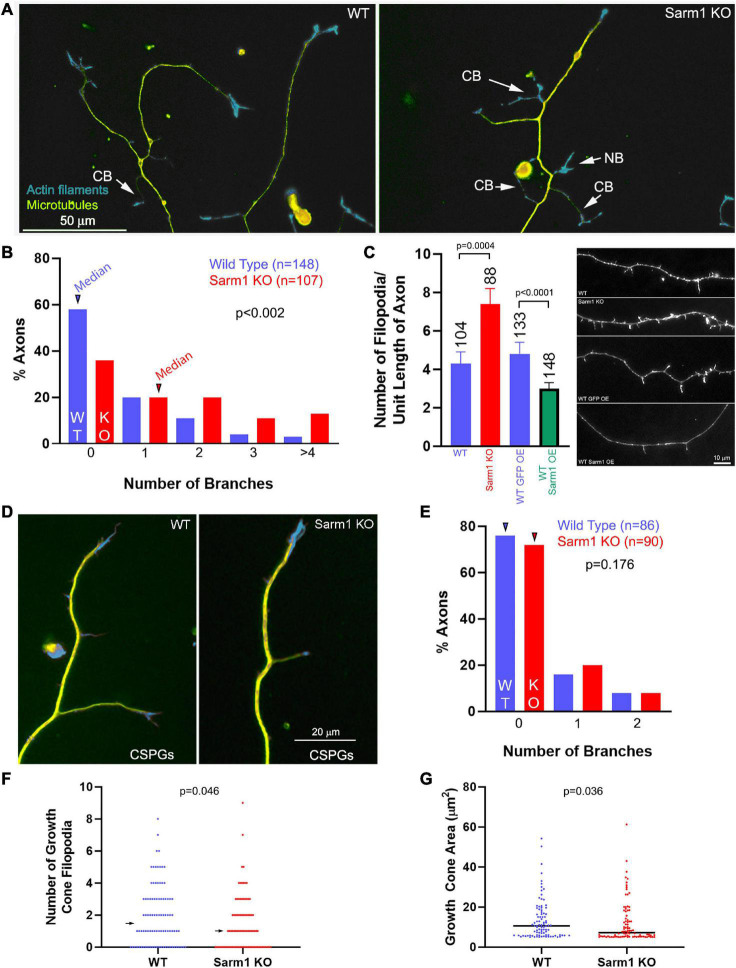
Axons of cultured SARM1 KO neurons exhibit increased branching. **(A)** Examples of axons of cultured dissociated wild-type (WT) and SARM1 knockout (KO) sensory neurons stained to reveal the actin filament (rhodamine phalloidin) and microtubule (anti-α-tubulin antibody) cytoskeleton. CB = collateral branches defined by the presence of a microtubule array and polarized distal actin filaments. NB = a nascent branch that is developing a distal growth cone. **(B)** Histogram showing the distribution of branch number along the axons of WT and SARM1 KO axons. Arrowheads denote the medians. **(C)** Graph showing the number of filopodia along the axons of WT and SARM1 KO (two leftmost bars) and those of WT expressing mitochondrially targeted GFP and overexpressing GFP-SARM1 (two rightmost bars). Representative examples of axonal morphology are shown on the right of the graph (phalloidin staining revealing actin filaments). **(D)** Examples of WT and SARM1 KO neuron distal axons cultured on CSPG substrata. **(E)** Histogram showing the distribution of branch number along the axons of WT and SARM1 KO axons cultured on CSPGs. Arrowheads denote the medians. **(F)** Number of filopodia in the growth cones of WT and SARM1 KO neurons. Arrows denote medians. **(G)** Measurement of the area of WT and SARM1 KO neuron growth cones. Black bars denote medians. A measurement of 5–6 μm^2^ reflects a growth cone with no lamellipodia, and the area is representative of the distal axon as specified in “Materials and Methods” section.

**FIGURE 2:**
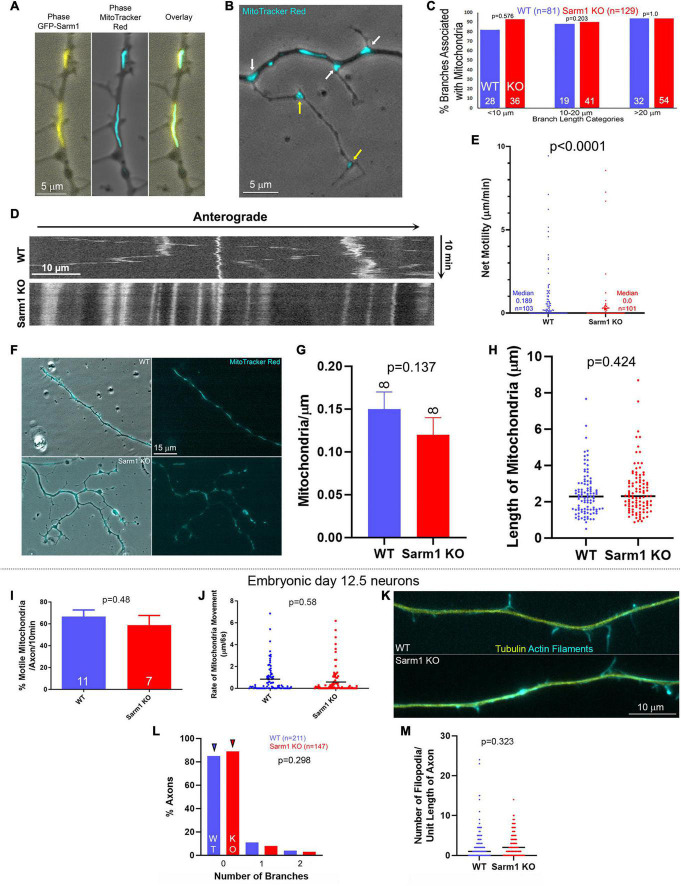
Mitochondria along the axons of SARM1 KO neurons exhibit decreased motility. **(A)** Example of a WT axon expressing GFP-SARM1 and mitochondria labeled using Mitotracker Red. GFP-SARM1 is targeted to mitochondria. **(B)** Examples of MitoTracker Red-labeled mitochondria targeted to the base of branches and filopodia (white arrows) of a SARM1 KO axon. Yellow arrows denote mitochondria in established branches. Phase contrast imaging is performed to show axonal morphology. **(C)** Histogram of the percentage of axonal protrusions with axonal mitochondria present at the base. Axonal protrusions are divided into three length classes. Those shorter than 10 μm reflect mostly filopodia and filopodia with some degree of distal polarization possibly reflective of the earliest stages of maturation into a branch. Those in the 10–20 μm bin reflect nascent branches, and those greater than 20 μm established branches with clear shafts and distal growth cones. **(D)** Kymograph of mitochondrial motility in WT and SARM1 KO axons. **(E)** Measurements of an index of net mitochondrial motility. The total summed displacement of a mitochondrion was determined during the entire imaging period regardless of directionality, and divided by the duration of the imaging period to obtain microns/min. Each datum reflects on a mitochondrion, sampled from 8 axons per group. Acquisition rate was every 6 s for 10 min. **(F)** Examples of WT and SARM1 KO axons with mitochondria labeled using MitoTracker red. **(G)** Density of mitochondria (mitochondria/μm) along the WT and SARM1 KO axons imaged. **(H)** Lengths of mitochondria along the WT and SARM1 KO axons imaged. Each datum reflects a mitochondrion. The black line denotes the median. **(I–M)** The data are representative of embryonic day 12.5 sensory neurons. **(I)** Percent of motile mitochondria per video sequence of embryonic sensory axons labeled with MitoTracker Red. n = number of axons imaged. **(J)** Rate of mitochondrial movement in either direction, from all the mitochondria sampled along the axons in **(I)**. A rate of zero reflects an immotile mitochondrion. The *p*-value is for comparison between the WT and SARM1 KO groups considering only mitochondria with rates greater than zero (i.e., the moving population). Black lines denote the medians. **(K)** Examples of the morphology of the axons of embryonic sensory neurons. **(L)** Histogram showing the distribution of branch number along the axons of embryonic WT and SARM1 KO neurons. Arrowheads denote the medians. **(M)** Number of filopodia per unit length along the axons of WT and SARM1 KO neurons. Black bars denote the medians.

Postnatal day 0–2 dissociated sensory neurons from wild-type (WT) or SARM1 KO (−/− throughout) mice were cultured on polylysine- and laminin-coated substrates. Cultures were then fixed and stained to reveal F-actin using phalloidin and tubulin using anti-α-tubulin antibodies. SARM1 KO neurons exhibited an increase in the number of branches per unit length of axon relative to the WT neurons (Figures 1A,B). Axonal filopodia are the first structures formed in the process of collateral branch formation. SARM1 KO axons exhibited an elevated number of axonal filopodia (Figure 1C). Conversely, the overexpression of SARM1 in WT neurons decreased the number of filopodia along distal axons (Figure 1C; see Figure 2A for an example of exogenously expressed SARM1 along axons). These data indicate that SARM1 is a negative regulator of the number of axonal filopodia and, subsequently, branches *in vitro*.

CSPGs suppress axon collateral branching along sensory axons *in vivo* and *in vitro* through suppression of PI3K-Akt signaling and mitochondrial respiration (Fisher et al., 2011; Silver et al., 2014; Sainath et al., 2017a,b). To assess whether SARM1 KO may promote branching on a branch formation inhibitory substratum, we analyzed the number of branches along sensory axons cultured on CSPGs and laminin relative to laminin alone (as in Sainath et al., 2017a). SARM1 KO did not elevate the number of axon branches on a CSPG substratum (Figures 1D,E). Thus, although SARM1 KO promotes branching on a permissive substratum, it fails to do so under conditions wherein a major upstream regulatory pathway (PI3K) is impaired.

We also considered whether SARM1 KO might affect growth cone morphology. Analysis of growth cone area and the number of growth cone filopodia revealed decreases in both metrics in the SARM1 KO group relative to WT (Figures 1F,G). The medians of growth cone area and number of filopodia were decreased 30 and 33% in SARM1 KO relative to WT, respectively. The effects of SARM1 KO on increase in branching and axonal filopodial number, thus, do not translate into similar effects on growth cones, which exhibit a decrease in surface area and number of filopodia relative to WT.

### SARM1 Knockout Cutaneous Sensory Endings in Skin Exhibit Increase in Terminal Branching

**FIGURE 3:**
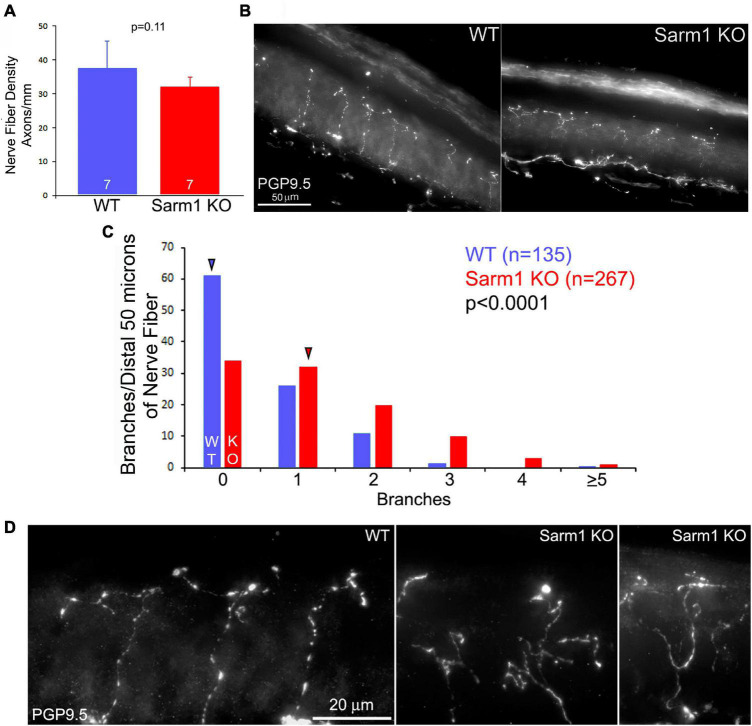
Terminals of SARM1 KO sensory cutaneous axons exhibit more complex types of morphology than those of WT axons *in vivo*. **(A)** Graph of the intra-epidermal nerve fiber density in WT and SARM1 KO skin. n = number of mice (120–127 days of age). **(B)** Examples of WT and SARM1 KO skin sections stained with anti-PGP9.5 used to obtain the intra-epidermal nerve fiber density measurements presented in **(A)**. **(C)** Histogram of the number of branches present along the distal 50 microns of sensory fibers. A value of 0 reflects a fiber with no branches and only its terminus. Arrowheads denote the medians. **(D)** Examples of the morphology of the termini of nerve fibers in WT and SARM1 KO samples.

As the *in vitro* data show an increase in the number of axon branches along distal sensory axons, we addressed whether a similar phenotype would be evident *in vivo* within the peripheral target tissue of the sensory neurons, the skin. We addressed two aspects of skin innervation, the density of axons innervating the skin and the extent of formation of branches by distal axons in the skin. Analysis of the innervation density of cutaneous sensory fibers/axons stained using antibodies against PGP9.5 in sections of footpad skin obtained from WT and KO adult mice did not show an increase in IENFD (Figures 3A,B), indicating that SARM1 does not have an impact on the targeting or retention of axons to the dermis. However, evaluation of the morphology of the distal 50 microns of nerve fiber endings, located within the stratum spinosum, showed increased complexity reflected by a greater number of distal branches (Figures 3C,D). Thus, both *in vitro* and *in vivo* distal sensory axons exhibit increased morphological complexity characterized by higher levels of branching.

### Axonal Mitochondria Exhibit Decreased Motility in SARM1 Knockout Postnatal Neurons

The positioning of stationary mitochondria along axons determines the sites of highest filopodium formation and branching along sensory and central nervous system axons (reviewed in Smith and Gallo, 2018), and in sensory neurons SARM1 is targeted to the outer mitochondrial membrane through an N-terminus sequence (Gerdts et al., 2013). SARM1 is, thus, localized to an organelle with a fundamental role in the formation of filopodia and branches. Consistent with prior determination in sensory neurons (Gerdts et al., 2013), exogenously expressed full-length WT SARM1 containing the N-terminus mitochondrial targeting sequence localized to mitochondria (Figure 2A). Analysis of the bases of axonal protrusions associated with mitochondria within the axon shaft did not reveal a difference between WT and SARM1 KO neurons (Figures 2B,C), indicating that mitochondrion positioning at the bases of branches is not affected, although SARM1 KO axons exhibit a greater number of branches. During the formation of branches along the axons of sensory neurons, axonal mitochondria are transported into branches and populate the nascent branches (Spillane et al., 2013). To assess whether the targeting or retention of mitochondria into nascent branches from the main axon shaft might differ between WT and SARM1 KO neurons, we analyzed the same population of axons, as shown in Figure 2C, and determined the proportions of branches that contained one or more mitochondria. This analysis showed that 59 and 77% of branches in the 10–20-μm length range and 100 and 98% in the > 20-μm length range contained mitochondria in the WT and SARM1 KO neurons, respectively (Fisher exact test, *p* = 0.203 and 1, respectively). These data indicate that the targeting of mitochondria at the base and into the branches does not differ among the genotypes, and that the relationship between positioning of mitochondria and their targeting into nascent branches is maintained along the axons of SARM1 KO neurons.

A prior study using embryonic mouse sensory neurons determined that SARM1 KO does not affect mitochondrial membrane potential, density, or the percentage of actively transported mitochondria along the axons of cultured mouse embryonic day 12.5 sensory neurons (Summers et al., 2014). In contrast, through live imaging analysis of the motility of mitochondria labeled using Mitotracker Red dye, we found that mitochondria along P0-2 SARM1 KO neuron sensory axons exhibit less net motility than those along WT axons (Figures 2D,E). Analysis of the proportion of mitochondria that remained stationary showed that 67% (*n* = 103) and 37% (*n* = 101) remained stationary during the imaging period along SARM1 KO and WT axons, respectively (Fisher’s exact test, *p* < 0.0001). Consideration of the motility of mitochondria initially associated with branches or filopodia at the beginning of imaging did not reveal a difference in the proportion of mitochondria that remained stalled in the initial position between WT and SARM1 KO (Fisher’s exact test, *p* = 0.369), indicating that the retention of mitochondria in branching/filopodial sites does not differ as a function of SARM1 KO and consistent with the prior analysis (Figures 2B,C). Analysis of the density of mitochondria per unit length of the axons or the length of mitochondria did not reveal any differences between WT and SARM1 KO (Figures 2F–H). Overall, the data indicate that although there are less motile mitochondria along the axons of SARM1 KO neurons, the association of mitochondria with sites of branching and filopodia and their retention in these sites does not vary between WT and SARM1 KO.

To address the differences in mitochondrial motility between the above data obtained from postnatal neurons and the study by Summers et al. (2014), we repeated the experiments using embryonic day 12.5 sensory neurons, the same developmental age as Summers et al. (2014) study. Consistent with Summers et al. (2014), we did not detect differences in mitochondrial motility within the axons of SARM1KO and WT embryonic sensory neurons (Figures 2I,J). Neither the proportion of mitochondria undergoing transport nor the distance they moved per unit time differed among the genotypes. Furthermore, the axons of SARM1 KO embryonic sensory neurons did not exhibit increased axon branching or number of filopodia along axons relative to WT (Figures 2K–M). Collectively, the data from embryonic and postnatal sensory neurons indicate that the branching phenotype of the SARM1 KO neurons is age-dependent and that the alterations in axonal mitochondrial motility observed in the postnatal neurons are correlated with increased axon branching.

### Axons of SARM1 Knockout Neurons Exhibit Increase in Actin Filament Dynamics Underlying the Formation of Axonal Filopodia

**FIGURE 4:**
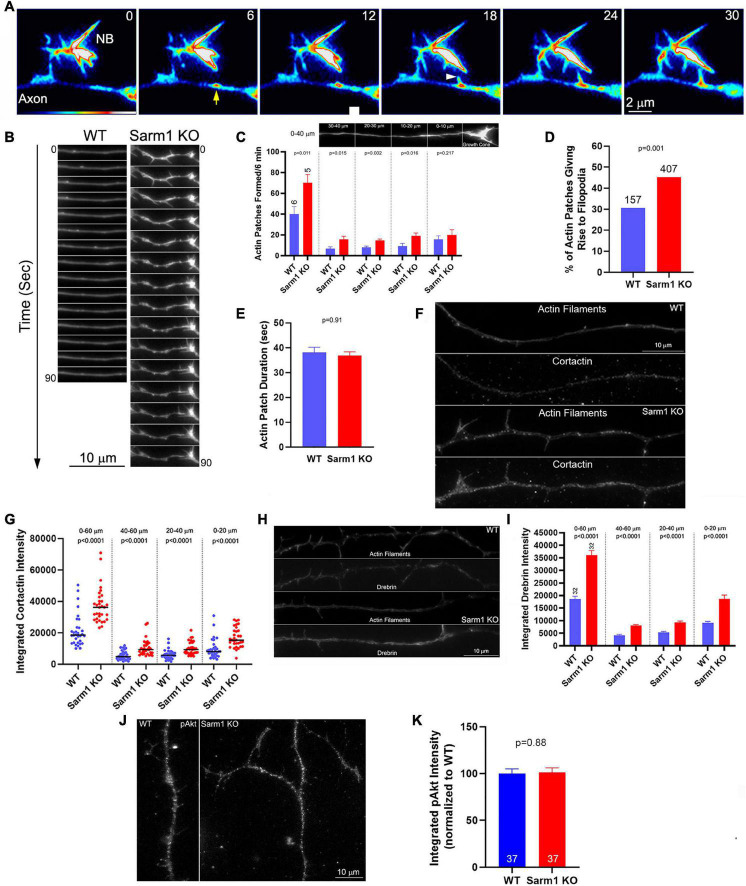
Axons of SARM1 KO neurons exhibit increased rates of actin patch formation and emergence of filopodia. **(A)** Example of actin patch formation and emergence of a filopodium from an actin patch as imaged using mCherry-β-actin. The axon segment (SARM1 KO) contains a nascent branch (NB) (numbers in panels denote seconds). At 6 s, an actin patch forms (yellow arrow). At 18 s, a filopodium emerges from the actin patch (white arrowhead) and subsequently elongates. **(B)** Qualitative examples of axonal actin dynamics along WT and SARM1 KO. The axons of SARM1 KO neurons exhibited more frequent fluctuations at local levels of actin filaments and formation of filopodia. **(C)** Quantification of the rate of actin patch formation along axons. Leftmost bars show the rates along the distal 0–40 μm of the axon (0 defined as the point along the axon just proximal to the base of the growth cone (see image of the axon in the panel). Analysis of the rate of patch formation along the distal 0–40 μm divided into 10 μm bins is shown in the four rightmost sets of bar graphs. n = number of axons. **(D)** Proportion of axonal actin patches that give rise to filopodia is elevated in SARM1 KO neurons relative to WT. **(E)** Duration of actin patches does not differ between SARM1 KO and WT axons. **(F)** Examples of cortactin staining levels along distal WT and SARM1 axons. **(G)** Graph showing the quantification of cortactin along distal axons in the format initially shown in **(C)**. Black bars denoted medians. **(H)** Examples of drebrin staining levels along distal WT and SARM1 axons. **(I)** Graph showing the quantification of drebrin along distal axons. **(J)** Examples of axons stained with antibodies to Akt phosphorylated at T308 (pAkt). **(K)** Quantification of the integrated intensity of pAkt staining along axons.

Formation of axonal filopodia, the first step in axon branching, is dependent on axonal actin filament dynamics. Axonal actin patches serve as precursors to the emergence of axonal filopodia and present as transient local accumulations of actin filaments along axons (Figure 4A). While axons generate many patches, only a fraction gives rise to filopodia before dissipating. To address whether axonal actin dynamics might be impacted by SARM1 KO, WT and KO neurons were transfected with mCherry-β-actin expression plasmid prior to culturing. Live imaging and analysis of axonal actin dynamics were performed as previously described (Loudon et al., 2006; Ketschek and Gallo, 2010; Spillane et al., 2012; Sainath et al., 2017a; Armijo-Weingart et al., 2019). As in our prior study, we determined the rate of patch formation (patches/unit length of axon/unit time), duration (s) of patches, and percentage of patches that gave rise to filopodia along the distal 40 μm of the axons, excluding the growth cones. SARM1 KO resulted in 75% increase in the rate of axonal actin patch formation along the distal 40 μm of axons (Figures 4B,C). As the rate of actin patch formation decreases along the axons with increase in distance from the growth cones (Ketschek and Gallo, 2010; Spillane et al., 2011), we also analyzed the rates of formation in 10-μm bins. The rates of formation were elevated in the 10–40 μm distal segment of the axons, where axon branching is most prominent, but not in the distal most 0–10 μm just proximal to the growth cones (Figure 4C). This observation is consistent with the lack of promotion of growth cone morphology in the SARM1 KO neurons (Figures 1F,G). SARM1 KO also increased the proportion of actin patches that gave rise to filopodia by 48% (Figure 4D). SARM1 KO did not alter the duration of actin patches (Figure 4E). These data indicate that SARM1 negatively regulates the formation of axonal actin patches and the emergence of filopodia from actin patches.

Drebrin and cortactin are actin regulatory proteins that are targeted to axonal actin patches, and their experimental overexpression promotes the formation of axonal filopodia and branches (Spillane et al., 2011, 2012; Ketschek et al., 2016). Drebrin is also targeted to the base of axonal filopodia and promotes the entry of microtubule tips into filopodia (Ketschek et al., 2016). Therefore, we sought to determine the levels of drebrin and cortactin along the WT and SARM1 KO sensory axons. Quantitative immunocytochemical analysis of the levels of these proteins (as in Spillane et al., 2011, 2012; Ketschek et al., 2016) revealed both to be elevated along SARM1 KO distal axons relative to WT (Figures 4F–I). Analysis of the levels of axonal drebrin and cortactin within 20-μm bins from 0 to 60 μm revealed differences between WT and SARM1 KO axons within each length bin, indicating that the levels are uniformly increased along the axons. The levels of both cortactin and drebrin were elevated in the 0–20 μm bin relative to the 20–40 and 40–60 μm bins along the axons of both WT and SARM1 KO neurons (multiple comparison tests, *p* < 0.001 for all comparison with the exception of cortactin WT 0–20 vs. 20–40, *p* < 0.01). The 20–40 and 40–60 μm bins did not differ in any of the within genotype comparison groups. The distal (highest)-proximal gradient in the levels of cortactin and drebrin along the distal axons is consistent with the similar gradient in the rate of actin patch formation rates (Ketschek and Gallo, 2010; Spillane et al., 2011) and was not altered as a function of genotype. The data, thus, indicate that the increase in the number of axonal filopodia along the axons of SARM1 KO neurons is due to increase in the rate of formation of axonal actin patches along the axons and the probability that filopodia will emerge from the patches, consistent with a prior study indicating that regulation of the rate of axonal actin patch formation is a major regulatory node in the mechanism of axonal filopodium formation (Ketschek and Gallo, 2010; Spillane et al., 2011, 2012, 2013; Ketschek et al., 2016; Sainath et al., 2017b). The increases in the axonal levels of cortactin and drebrin are consistent with the concerted regulation of the actin regulatory mechanisms that underlie the promotion of actin patch formation and filopodium emergence from patches (Spillane et al., 2011, 2012, 2013).

The levels of axonal cortactin and branching of axons are positively regulated by the levels of PI3K-Akt signaling along the sensory axons (Ketschek and Gallo, 2010; Spillane et al., 2012). Thus, we assessed whether the levels of activated Akt, phosphorylated in the primary activating site of threonine 308, might be elevated along the axons of SARM1 KO neurons using previously published quantitative immunocytochemical methods (Sainath and Gallo, 2021). The axons of SARM1 KO neurons did not exhibit changes in levels of activated Akt (Figures 4J,K). These data are generally consistent with a prior study assessing that SARM1 does not regulate axon degeneration through regulation of Akt (Yang et al., 2015). Thus, the increase in levels of cortactin and axon branching along the axons of SARM1 KO neurons are not readily attributable to alterations in Akt signaling.

### Axonal Filopodia of SARM1 Knockout Neurons Are More Dynamic

**FIGURE 5:**
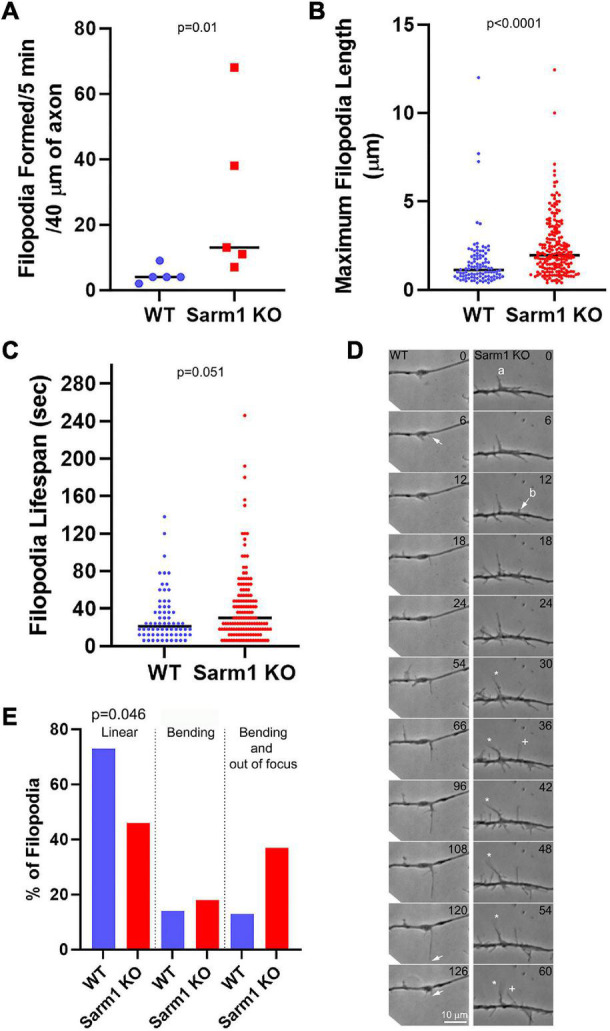
Axons of SARM1 KO neurons exhibit altered filopodium dynamics. **(A)** Rate of filopodium formation is elevated along SARM1 KO axons relative to WT. Analysis in this Figure is performed from phase contrast time lapse videos. Each datum represents one axon. **(B)** SARM1 KO filopodia attain longer maximal lengths. Each datum represents a single filopodium from the axons in **(A)**. **(C)** SARM1 KO filopodia exhibit longer lifespans. Each datum represents a single filopodium from the axons in **(A)**. **(D)** Examples of filopodial dynamics along WT and SARM1 KO axons (time in s). A WT filopodium emerged from an axon shaft (arrow at 6 s) and elongated to its maximal length (arrow at 120 s) before being retracted (arrow at 126 s). During the imaging period, the shaft of the filopodium remained in the imaging plane and did not exhibit overt swinging/bending. In contrast, the filopodia of SARM1 KO neurons exhibited greater dynamics after emergence. Two filopodia are discussed and labeled a and b. Between 30 and 60 s, the tip of filopodium a goes out of the focal plane, indicating upward movement from the substratum and bends to the left (denoted by the white star symbol). Filopodium b emerges from the shaft at 12 s (arrow) and elongates until 36 s when its tip also exits the imaging plane (denoted by +). Between 54 and 60 s, the shaft of filopodium b bends to the left (denoted by + at 60 s). **(E)** Graph showing the percentage of axonal filopodia that exhibited linear extension and retraction remaining within the plane of focus and not bending (linear), filopodia that remained within the plane of focus but underwent bending (bending), and filopodia that underwent bending and had the tip exit the plane of focus (bending and out of focus) (chi squared test comparing the linear vs. the combined non-linear categories across genotype).

We next determined whether SARM1 KO might have an impact on aspects of filopodial dynamics after emergence by phase contrast optics. Consistent with the data from imaging actin dynamics (Figure 4), the axons of SARM1 KO neurons exhibited a higher rate of filopodium formation than those of WT neurons (Figure 5A). The median maximum length attained by filopodia increased by 73% along the KO axons (Figure 5B). Measurement of the lifespan of newly formed filopodia revealed that KO filopodia exhibited a 42% increase in median duration from the time of emergence to that of complete retraction into the axons (Figure 5C). Although the filopodia of KO axons exhibited longer life spans, the proportion of filopodia with lifespans greater than 60 s within genotype was not different between WT (25%) and SARM1 KO axons (28%) (Figure 5C), indicating that SARM1 KO does not increase the stabilization of filopodia for prolonged time periods (Fisher’s exact test, *p* = 0.684).

Along the WT axons, filopodia initially emerge, and then their tips elongate in a linear manner remaining in close contact with the substratum until undergoing retraction back into the axon shaft (Figure 5D). In contrast, the filopodia of KO neurons exhibited a variety of behaviors including elevation from the substratum, buckling, and occasional collapsing upon themselves (Figure 5D) and 54% of the KO filopodia exhibited one or more of these behaviors in contrast to the 27% of WT (Figure 5E). The behavior of KO filopodia precluded an analysis of the rates of filopodial elongation and retraction, as the tip was not always available for tracking because of its movement out of the focal plane, thus, exclusion of filopodia that underwent these heightened dynamics from the sample would have skewed sampling. We note the caveat that maximal length was determined until the point filopodia exited the focal plane or if they returned to it and were longer, but this would result in underestimation of the length if a filopodium had extended further while out of the focal plane. However, even with this caveat, the filopodia extended to detectably longer lengths. The increase in the dynamics of axonal filopodia observed along the axons of SARM1 KO neurons might also contribute to the formation of axon branches by impacting the stage of branch maturation wherein actin dynamics are increased in filopodia that mature into branches and reorganize the filaments to attain a distally polarized distribution, but this issue will require additional consideration.

### Axons of SARM1 Knockout Neurons Exhibit Increase in Microtubule Plus Tip Dynamics

**FIGURE 6:**
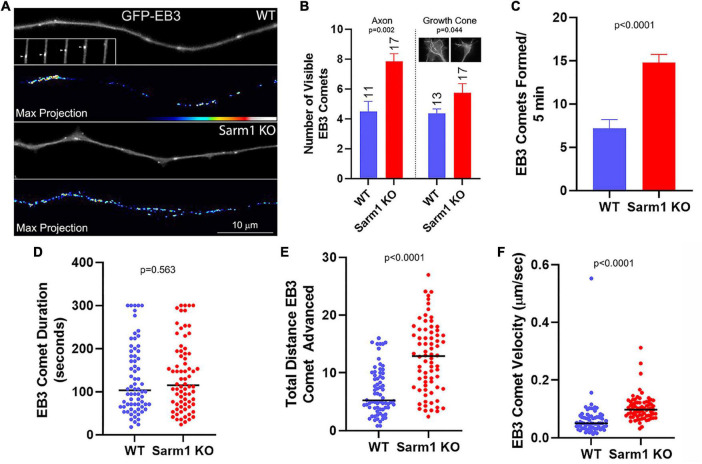
Axons of SARM1 KO neurons exhibit increased microtubule plus tip dynamics. **(A)** Examples of GFP-EB3 comets in axons. The GFP-EB3 panels represent the first frame in a 5- min video sequence. The inset in the WT GFP-EB3 panel tracks a representative single comet (arrowhead) advancing in consecutive 6-s frames (left to right) until it dissipates. The panels labeled Max Projection show maximal intensity projection of the entire Z-stack of the timelapse. The false colored max projections, thus, depict the integrated number and location of all comets during the video providing a qualitative at-a-glance appreciation of the net levels of comets observed throughout the video. **(B)** Graph of the number of comets visible along the distal 0–50 μm of the axon, and separately in the growth cone, sampled at 30-s intervals, representing a gross measure of net comet number per axon. The insets in the growth cone subpanel show max projections of growth cones, as for the axons in **(A)**. n = number of axons and the sample was used for the rest of the analysis in this figure. **(C)** Graph of the rate of EB3 comet formation along distal axons. **(D)** Graph of EB3 comet duration (time from emergence to dissipation). In WT and SARM1 KO axons 7 and 5.4% of the comets lasted for the duration of the timelapse sequence (300 s), respectively. **(E)** Graph of the total distance advanced by comets spanning their duration. **(F)** Graph of the velocity of EB3 comet advance.

The entry of microtubule plus tips into axonal filopodia is required for a filopodium to mature into a branch. Therefore, given the increase in branching observed along the SARM1 KO axons, we addressed whether axonal microtubule plus tip behavior may also be impacted. Microtubule plus tip polymerization can be tracked using fluorescently tagged end binding protein 3 (EB3; Stepanova et al., 2003). EB3 is recruited to a plus tip when the tip begins to polymerize and, when imaged, presents as a “comet” at the tip of a microtubule (Figure 6A). To obtain a general metric of the prevalence of comets in axons and growth cones, we counted the number of comets per axon and growth cones present in timelapse frames at 30-s intervals. There was a 68% increase in the mean number of GFP-EB3 comets along the distal axons (Figures 6A,B). The mean number of comets was also increased in the growth cones although by only 32% (Figure 6B), slightly less than half of the increase along the distal axons. In order to obtain additional insights, we analyzed multiple parameters of individual EB3 comet dynamics. The rate of new comet formation along the distal 50 microns of axons was approximately doubled in the SARM1 KO axons relative to those of the WT (Figure 6C). Although the duration (time from formation to dissipation) of comets did not differ (Figure 6D), the total distance individual comets advanced within the axons during their duration was increased in the SARM1 KO axons (Figure 6E) because of increased comet advance velocity (Figure 6F). The axons of SARM1 KO neurons, thus, exhibit a greater probability of comet formation and a greater velocity of comet advance, reflective of the rate of microtubule plus tip polymerization.

**FIGURE 7:**
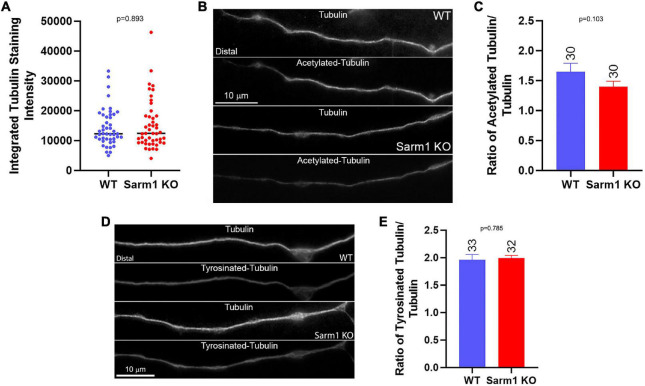
SARM1 KO has no impact on the levels of axonal microtubules and acetylated and tyrosinated tubulin. **(A)** Graph of total microtubule mass determined by the integrated staining intensity of α-tubulin along distal axons that were simultaneously fixed and extracted to retain polymeric tubulin (microtubules) and remove soluble tubulin, a method used through the figure. **(B)** Examples of total microtubule (α-tubulin) and acetylated tubulin staining along the axons of WT and SARM1 KO neurons. **(C)** Graph of the ratio of acetylated to total tubulin staining intensity along distal axons. **(D)** Examples of total microtubule (α-tubulin) and tyrosinated tubulin staining along the axons of WT and SARM1 KO neurons. **(E)** Graph of the ratio of tyrosinated to total tubulin staining intensity along distal axons.

As in the prior study, to measure the net levels of axonal microtubules, we used cultures that had been simultaneously fixed and permeabilized to remove soluble tubulin while retaining polymerized microtubules (e.g., Gallo and Letourneau, 1998). Measurement of the total staining intensity of α-tubulin, reflective of microtubules, in the distal 40 μm of axons did not show a difference in net microtubule mass (Figures 7A,B,D). Tubulin is extensively post translationally modified. Following polymerization into the microtubule lattice, α-tubulin undergoes detyrosination and acetylation in a time-dependent manner (Janke and Magiera, 2020). Quantification of the ratio of acetylated and tyrosinated tubulin to total microtubule levels using fixed and permeabilized samples, as described above, did not show changes in the ratio of post translational modification between WT and SARM1 KO axons (Figures 7B–E). The differences in axonal plus tip dynamics between WT and SARM1 KO, thus, do not translate into altered net levels of microtubules or into the extent of acetylation or tyrosination of α-tubulin. The observed increases in axonal microtubule plus tip dynamics likely contribute to the promotion of axon branching along the axons of SARM1 KO neurons by promoting the targeting of microtubule plus tips into axonal filopodia, a required component of the formation of branches from filopodia.

## Discussion

Axon collateral branching is regulated at multiple levels through the dynamics of the axonal cytoskeleton. Increases in the formation of filopodia promote the formation of branches at the first step in the mechanism. Similarly, increases in microtubule plus tip polymerization positively contribute to axon branching by increasing the availability of microtubule tips that can be targeted into axonal filopodia and initiate the process of maturation of a filopodium into a branch. This study demonstrates that genetic loss of SARM1 in sensory neurons results in increased branching, likely a consequence of the promotion of actin filaments and microtubule dynamics underlying the process of branching. The increase in filopodium formation is attributable to a concerted increase in the rate of formation of axonal actin patches, precursors to filopodial emergence, and an increase in the proportion of actin patches that give rise to filopodia. An increase in microtubule plus tip polymerization would result in increased tips available for entry into the base of axonal filopodia and, thus, increase the probability of a filopodium maturing into a branch.

In this study we observed that the axons of postnatal SARM1 KO sensory neurons exhibited less mitochondrial motility than the WT axons. The decrease in mitochondrial motility along the axons of postnatal SARM1 KO neurons is consistent with the observed increases in branching, as stationary mitochondria determine sites where filopodia and branches preferentially form along axons (reviewed in Smith and Gallo, 2018). The increase in stationary mitochondria along the SARM1 KO axons suggests an explanation for at least one contribution of SARM1 KO to the increase in branching observed in the postnatal KO neurons; increasing the proportion of stationary mitochondria, therefore, results in increased sites of filopodia and branch formation along axons. Consistent with the report by Summers et al. (2014), which did not find an effect of SARM1 KO on mitochondrial motility along embryonic sensory axons, we similarly did not detect differences in mitochondrial motility along the axons of embryonic sensory neurons. Furthermore, the axons of embryonic sensory SARM1 KO neurons did not exhibit increased branching. Jointly, the data from postnatal and embryonic sensory neurons indicate a correlation between decreased axonal mitochondrial motility and increased axon branching. Additional studies will be required to determine experimentally through manipulation of mitochondrion-stalling mechanisms along axons whether the relationship is causal. Consistent with the neuron’s age being a potential contributing factor to the differences between embryonic and postnatal sensory neurons, as sensory neurons age they develop increased number of branches upon culturing, an effect reversed by a prior lesion to the axons that reverts to the neurons to a more immature mode of axon extension (Smith and Skene, 1997). Similarly, the number of stationary mitochondria along axons increases with the age of neurons (Lewis et al., 2016), in concert with increased expression levels of the mitochondrion-microtubule-anchoring protein syntaphilin (Zhou et al., 2016). The length of axonal mitochondria increases with the developmental age of sensory neurons (Armijo-Weingart et al., 2019), and the transport of axonal mitochondria is inversely proportional to the length of mitochondria (Misgeld et al., 2007; Narayanareddy et al., 2014). Thus, older neurons may be more prone to suppression of mitochondrial motility in the context of SARM1 KO and more predisposed toward the formation of axon branches. The mechanism through which SARM1 is impacting mitochondrial motility remains to be addressed. As SARM1 is targeted to the outer mitochondrial membrane (Gerdts et al., 2013), it is poised to interfere or modulate aspects of the mechanism of active transport at the level of motor proteins or adaptors (Schwarz, 2013; Smith and Gallo, 2018). Similarly, SARM1 may be modulating aspects of the microtubule-based mechanism of mitochondrial stalling (Zhou et al., 2016). Alternatively, SARM1 KO may also regulate the expression levels of proteins involved in mitochondrial transport or anchoring. Whether SARM1 KO has an impact on the transport of additional axonal cargoes remains to be addressed.

The axons of SARM1 KO neurons contained higher levels of the actin regulatory and collateral branch formation promoting-proteins drebrin and cortactin (Spillane et al., 2011, 2012; Ketschek et al., 2016). The current experiments were performed in the presence of neurotrophins. NGF induces intra-axonal translation of cortactin (Spillane et al., 2012; Ketschek et al., 2016), and axonal translation occurs preferentially at sites demarked by stalled mitochondria (Spillane et al., 2013; Sainath et al., 2017a; Wong et al., 2017). The increase in the proportion of stalled mitochondria may, thus, also be contributing to the increases in levels of cortactin by increasing sites of localized translation along axons. In contrast, NGF increases the levels of axonal drebrin in a manner independent of translation (Ketschek et al., 2016). The increases in protein levels of cortactin and drebrin may also be due to decreased proteolysis, increased protein stability, increased transport into axons, or increased somatic transcription and/or translation. Future studies will be required to address these alternative but not mutually exclusive hypothetical mechanisms for the regulation of the axonal levels of cortactin and drebrin by SARM1 KO. Consistent with the possible role of SARM1 as a negative regulator of axonal translation, Izadifar et al. (2021) recently reported that SARM1 may impede the synthesis of axon-protective proteins such as Nmnat.

The axons of SARM1 KO neurons exhibited elevated microtubule plus tip dynamics reflected in increased rates of comet formation and faster velocities of comet advance. This difference in dynamics may contribute to the increase in branching by providing a greater density of actively polymerizing tips within axons that may then undergo targeting into axonal filopodia. Drebrin binds actin filaments at the base of filopodia and promotes targeting of microtubule plus tips into axonal filopodia, thereby contributing to the microtubule-based component of the mechanism of branching (Ketschek et al., 2016). The increase in drebrin levels within SARM1 KO axons is, thus, expected to promote the entry of microtubule tips into axonal filopodia in concert with the increase in plus tip dynamics. As there is not a detectable increase in net microtubule mass along axons, the increase in plus tip dynamics may be counterbalanced by increase in catastrophes (when a tip undergoes a depolymerization event) or increase in mass below our detection range. Unfortunately, EB3 imaging does not allow for the analysis of microtubule depolymerization events, and because of the compact nature of the axonal microtubule array, individual microtubules in their entirety cannot be imaged live. The issue of whether there may be a concerted increase in tip depolymerization dynamics to counterbalance the increase in plus tip polymerization remains to be addressed. Vincristine is a microtubule-depolymerizing agent that is used at low doses as a cancer therapeutic but results in peripheral neuropathy. SARM1 KO counters the effects of vincristine on peripheral nerves (Geisler et al., 2016). The effects of SARM1 KO on microtubule plus tip polymerization reported in this study may, at least in part, underlie the effects of SARM1 KO on vincristine neuropathy due to increase in actively polymerizing plus tips in axons in addition to the established role of SARM1 in the activation of the axonal degeneration mechanism. Although tubulin post-translational modifications of acetylation and tyrosination can serve as indirect reporters of net microtubule dynamics, they are regulated by upstream enzyme systems that are, in turn, regulated by additional mechanisms (Janke and Magiera, 2020). This study finds that increased plus tip dynamics do not correlate with levels of tubulin acetylation and tyrosination, which would be predicted to be decreased and increased, respectively, in the context of greater microtubule dynamics. It is, thus, possible that SARM1 KO may also be affecting the systems regulating these post-translational modifications, but this remains to be determined. Consistent with this notion, Chen et al. (2011) found that SARM1 levels negatively regulate the ratio of acetylated to total tubulin in COS-1 and neuro-2A cells. Alternatively, the effects of SARM1 KO on microtubules may be below the threshold to result in detectable changes in acetylation and tyrosination, or, as noted above, are countered by increases in catastrophes, thereby maintaining the microtubule array at WT levels of microtubule mass and levels of post-translational modifications.

The data indicate that, minimally, SARM1 KO promotes the formation of branches at the earliest steps in the mechanism, and the formation of axonal filopodia and axonal microtubule dynamics. However, future studies will be needed to determine if it might also have an impact on aspects of the maturation of filopodia into nascent branches and the subsequent stability of branches in varying stages of their development. Whether the effects of SARM1 KO on axonal shaft microtubule plus tips are also present within nascent branches was not determined in this study. As SARM1 is associated with mitochondria, and mitochondria are targeted normally to both the bases and within the shafts of branches, it is likely that microtubules within branches are also affected by SARM1 KO. MAP7 is a microtubule-associated protein that promotes branch stabilization by impacting microtubule depolymerization and modulating organelle transport (Tymanskyj et al., 2018; Tymanskyj and Ma, 2019). Whether MAP7 level or function may be altered in SARM1 KO neurons remains to be determined.

Inhibition of SARM1 is a potential therapeutic for axon degeneration and neuronal cytotoxicity (Gerdts et al., 2016; Figley and DiAntonio, 2020). This study indicates that inhibition of SARM1 may also be able to manipulate the degree of axon collateral branching, often referred to as sprouting, in injury contexts. Injury-related axon sprouting can have beneficial or adverse effects on plasticity following a nervous system injury (Onifer et al., 2011). Inhibition of SARM1 may, thus, be worth considering in injury contexts wherein sprouting is beneficial, and similarly as potentially having an adverse “side-effect” if in a treatment context it may induce maladaptive sprouting.

SARM1 KO had more pronounced effects on axon shafts than on growth cones. Prior studies have also indicated that the growth cone and the axon shaft of sensory neurons are differentially regulated. For example, mitochondria exhibit more hyperpolarized potentials in growth cones of sensory neurons than along axon shafts (Verburg and Hollenbeck, 2008), and the elaboration of sensory neuron growth cones in response to NGF is not dependent on localized translation while the elaboration of axon branches and the underlying promotion of actin cytoskeletal dynamics are dependent on axonal translation (Roche et al., 2009; Spillane et al., 2012). We did not observe a promotion in growth cone morphology due to SARM1 KO, and the effects on actin patch formation along the axon shafts were not evident in the distal most 10 μm of the axons in proximity to the growth cones. Although EB3 comets were increased in the growth cones of SARM1 KO neurons, the relative increase was only about half of that observed along the axon shafts. As we did not perform an exhaustive analysis of the growth cones, there could be aspects of growth cone biology affected by SARM1 KO that may be unveiled by future investigations. It is possible that under the conditions of our study, performed on growth-promoting laminin and in the presence of neurotrophins, the growth cone is already at a maximal response level and, thus, cannot be further elaborated. If so, then SARM1 KO may have effects on growth cones in other environments such as growth inhibitory substrata or on mediating the response to growth cone collapsing signals.

In conclusion, this study determines that SARM1 KO results in increased axon collateral branching in postnatal sensory neurons, and that *in vivo* cutaneous SARM1 KO sensory endings have more complex types of morphology. Whether the *in vivo* phenotype may result in altered skin sensation or pain remains to be determined. Also, whether SARM1 KO will affect the branching morphogenesis of neuronal populations other than sensory neurons remains to be determined. However, a generalized role of SARM1 in negative regulation of axon morphology is suggested by the recent demonstration of a role of SARM1 in branching of mechanosensory neurons in Drosophila (Izadifar et al., 2021). The data presented herein provide a link between SARM1 and the suppression of the dynamics of both the axonal actin filament and microtubule cytoskeletons. The mechanisms linking SARM1 to cytoskeletal dynamics is a focus of ongoing studies. The observation that SARM1 fails to promote branching on branch-suppressing CSPGs, which attenuate an early signaling step in branch formation (PI3K-Akt activity), indicates that the effects of SARM1 are likely downstream of PI3K-Akt or other pathways impacted by CSPGs (Fisher et al., 2011). Consistently, Yang et al. (2015) did not find a role of SARM1 in the regulation of PI3K-Akt, but rather Akt suppresses MAPK activity that otherwise promotes SARM1-regulated axon degeneration (Walker et al., 2017), and in this study, we did not observe altered levels of Akt activation along the axons of SARM1 KO neurons. However, an axon injury activates MAPK, and SARM1 KO suppresses this activation (Yang et al., 2015), supporting the notion that SARM1 KO can have an impact on the function of signaling pathways, noting that the MAPK pathway is also activated by growth factors. The microtubule cytoskeleton is under regulation by a multitude of kinases such as those involved in mediating SARM1-dependent axon degeneration (Mandelkow et al., 1995; Bogoyevitch and Kobe, 2006) and may, thus, be regulated through these signaling pathways. Although SARM1 KO was not found to affect the levels of activated Akt along axons, it may be fruitful to further determine if SARM1 may regulate other aspects of the intracellular signaling of extracellular signals that induce axon branching, such as NGF.

## Data Availability Statement

The raw data supporting the conclusions of this article will be made available by the authors, without undue reservation.

## Ethics Statement

The animal study was reviewed and approved by the Institutional Animal Care and Use Committee, Lewis Katz School of Medicine.

## Author Contributions

GG designed the research, analyzed the data, and prepared the manuscript. AK and SH designed the research, conducted the research, analyzed the data, and revised the manuscript. All authors contributed to the article and approved the submitted version.

## Conflict of Interest

The authors declare that the research was conducted in the absence of any commercial or financial relationships that could be construed as a potential conflict of interest.

## Publisher’s Note

All claims expressed in this article are solely those of the authors and do not necessarily represent those of their affiliated organizations, or those of the publisher, the editors and the reviewers. Any product that may be evaluated in this article, or claim that may be made by its manufacturer, is not guaranteed or endorsed by the publisher.
